# *APOE* ε4 and Decline in Health and Financial Literacy in Advanced Age

**DOI:** 10.1111/jgs.70291

**Published:** 2025-12-30

**Authors:** Christopher C. Stewart, Lei Yu, Alifiya Kapasi, David A. Bennett, Patricia A. Boyle

**Affiliations:** ^1^ Department of Neurology Indiana University School of Medicine Indianapolis Indiana USA; ^2^ Rush Alzheimer's Disease Center Rush University Medical Center Chicago Illinois USA; ^3^ Department of Neurological Sciences Rush University Medical Center Chicago Illinois USA; ^4^ Department of Pathology Rush University Medical Center Chicago Illinois USA; ^5^ Department of Psychiatry and Behavioral Sciences Rush University Medical Center Chicago Illinois USA

**Keywords:** aging, Alzheimer's disease, *APOE*, literacy

## Abstract

**Background:**

Health and financial literacy decline in aging, but it is unclear why. In this study, we hypothesized that older people who are carriers of the *APOE* ε4 allele exhibit a steeper decline in literacy over time.

**Methods:**

Participants were 851 community‐dwelling older adults without dementia at analytic baseline (188 ε4 carriers and 663 noncarriers). Literacy was assessed at baseline and each year thereafter for up to 14 years.

**Results:**

In a linear mixed‐effects model adjusted for age, gender, and education, ε4 was associated with a lower starting level of literacy (*b* = −3.60, SE *b* = 1.00, *p* < 0.001) and, critically, a roughly 40% steeper decline in literacy over time (*b* = −0.41, SE *b* = 0.14, *p* = 0.004). The association between ε4 and literacy decline persisted after adjusting for global cognition at baseline (*b* = −0.35, SE *b* = 0.14, *p* = 0.012) and among a subgroup of participants with no cognitive impairment at baseline (*b* = −0.34, SE *b* = 0.14, *p* = 0.016).

**Conclusions:**

ε4 contributes to literacy decline among older adults, presumably due in part to the accumulation of neuropathologies associated with ε4. We discuss the potential clinical implications of ε4‐related literacy decline.

## Summary

- Key points
  ○ Among older adults without dementia at baseline, carriers of the *APOE* ε4 allele had a lower starting level of health and financial literacy and, more importantly, an approximately 40% steeper decline in literacy over time compared to noncarriers.
  ○ The association of ε4 with literacy decline was relatively independent of baseline cognition.
  ○ This study provides evidence against the idea that literacy usually remains stable in late life.
- Why does this paper matter?
  ○ To learn as much as they can about their risk of Alzheimer's dementia, older adults increasingly seek out *APOE* genotyping. With this, now more than ever, clinicians need to understand the broader implications of *APOE* ε4 positivity. Expanding upon the literature on ε4 and cognitive decline, in this paper, we report a novel association of ε4 with accelerated decline in health and financial literacy, a resource that is central to health and well‐being in aging.

## 1 Introduction

Health and financial literacy (hereafter called literacy) refers to the ability to engage with, learn about, and apply health and financial knowledge. Literacy is a linchpin of well‐being and independent living [1, 2]. Simply put, it is virtually impossible to navigate society without a basic understanding of healthy and unhealthy behaviors, common medical conditions, health insurance, mortgages, taxes, retirement plans, and so forth. In older adulthood, literacy holds extra significance. When health is on the decline and income is fixed, neither the body nor bank account rebound from missteps like they once did. Indeed, low literacy in advanced age has been linked to a wide range of adverse outcomes, including poorer physical and mental health [3, 4], financial exploitation [5], debt and bankruptcy [6, 7], and mortality [8].

While literacy is often thought of as fixed throughout adulthood, emerging evidence indicates that older adults' literacy declines over time [9, 10]. Notably, however, the vast literature linking low literacy to adverse outcomes has almost always examined literacy at a single timepoint, and this data point reflects both older adults' lifelong level of literacy and any literacy decline that preceded its measurement. Illustrating the importance of partitioning these components, our group recently associated steeper literacy decline with poorer healthcare and financial decision making, higher scam susceptibility, and lower psychological well‐being, independent of starting level of literacy [11]. This line of research, while nascent, has potential clinical importance. For example, the determinants of lifelong low literacy versus literacy decline will almost certainly differ, and this will impact the factors literacy interventions target. Moreover, if we can identify individuals at high risk of insidious literacy decline, then early action may mitigate or prevent subsequent hardship.

To investigate this topic, in this study, we hypothesized that the *apolipoprotein E* (*APOE*) ε4 allele is associated with a steeper decline in literacy in advanced age. We focused on ε4 because it is the chief genetic risk factor of Alzheimer's dementia [12] and because older adults increasingly pursue *APOE* genotyping. In prior cross‐sectional work, we reported an association of ε4 with lower literacy among older participants without dementia from the Rush Memory and Aging Project (MAP) [13]. This finding, however, does not disentangle starting level of literacy from literacy decline. Here, we leveraged MAP's longitudinal design to examine the relation of ε4 status with up to 14 years of annual literacy assessments. Because we were interested in early changes in literacy, in supporting analyses, we examined the association between ε4 and literacy decline after adjusting for a robust measure of global cognition at baseline and among a subgroup of participants with no cognitive impairment at baseline (i.e., no MCI or dementia).

## 2 Methods

### 2.1 Participants

MAP is an ongoing, clinical‐pathological study of aging [14]. Participants are community‐based older adults without known dementia at study entry who agree to annual clinical evaluations and organ donation. Alzheimer's dementia is assessed annually in MAP using the criteria of the National Institute of Neurologic and Communicative Disorders and Stroke and the Alzheimer's Disease and Related Disorders Association [15]. Participants with cognitive impairment but who do not meet criteria for dementia are diagnosed with MCI [16].

MAP began in 1997. A substudy of decision making, which includes an assessment of literacy, was added to MAP in 2010. Of the 1392 participants who completed a baseline decision making assessment, 74 were diagnosed with dementia at baseline; 297 had *APOE* genotype data pending; 2 had missing literacy data; and 168 had not completed a follow‐up decision making assessment. This left 851 participants eligible for this study.

MAP and the decision making substudy were approved by the Institutional Review Board of Rush University Medical Center. Written informed consent and a document of anatomical gift were obtained prior to study participation.

### 2.2 Health and Financial Literacy

The literacy measure was administered at analytic baseline and annually thereafter. It consisted of 32 items, of which 9 assessed health literacy and 23 assessed financial literacy. Items required utilization of health and financial information (e.g., heart disease, medical risk, stocks, compound interest) and were knowledge‐based (19 items) or required simple mental calculations (13 items) (the full measure can be found here [17]). Percent correct was calculated separately for health literacy and financial literacy and then averaged to yield a measure of total literacy. In prior work, we associated lower performance on this literacy measure with a range of negative behaviors and outcomes, including fewer health‐promoting behaviors [18], greater impairment in complex activities of daily living [18], worse diabetes indicators [19], and increased risk of hospitalization [20] and mortality [21].

### 2.3 *APOE* Genotyping

DNA was extracted from peripheral blood or frozen postmortem brain tissue. *APOE* genotype was determined by sequencing rs429358 (codon 112) and rs7412 (codon 158) at exon 4 of the *APOE* gene on chromosome 19. Genotyping was performed by investigators blinded to all clinical and pathologic data.

### 2.4 Statistical Analysis

Associations of *APOE* ε4 status with starting level of literacy (i.e., literacy at analytic baseline) and subsequent change in literacy were examined via linear mixed‐effects models, with annual total literacy scores as the longitudinal continuous outcome. The core model was comprised of terms for the intercept (i.e., literacy at analytic baseline), time in years since baseline, age at analytic baseline, gender, education, and ε4 status, as well as the interactions of age, gender, education, and ε4 status with time. The main effects measured the association of the terms with literacy at analytic baseline, and the interactions with time measured the association of the terms with change in literacy. In supporting analyses, we reran the core model, this time adding terms for global cognition at analytic baseline and the interaction of global cognition with time. Global cognition was measured via 19 neuropsychological tests that were converted to *z* scores based on the entire MAP cohort and then averaged [14]. Finally, we reran the core model among those participants with no cognitive impairment at analytic baseline (i.e., no MCI or dementia). Model results are reported as unstandardized β coefficients.

## 3 Results

**FIGURE 1:**
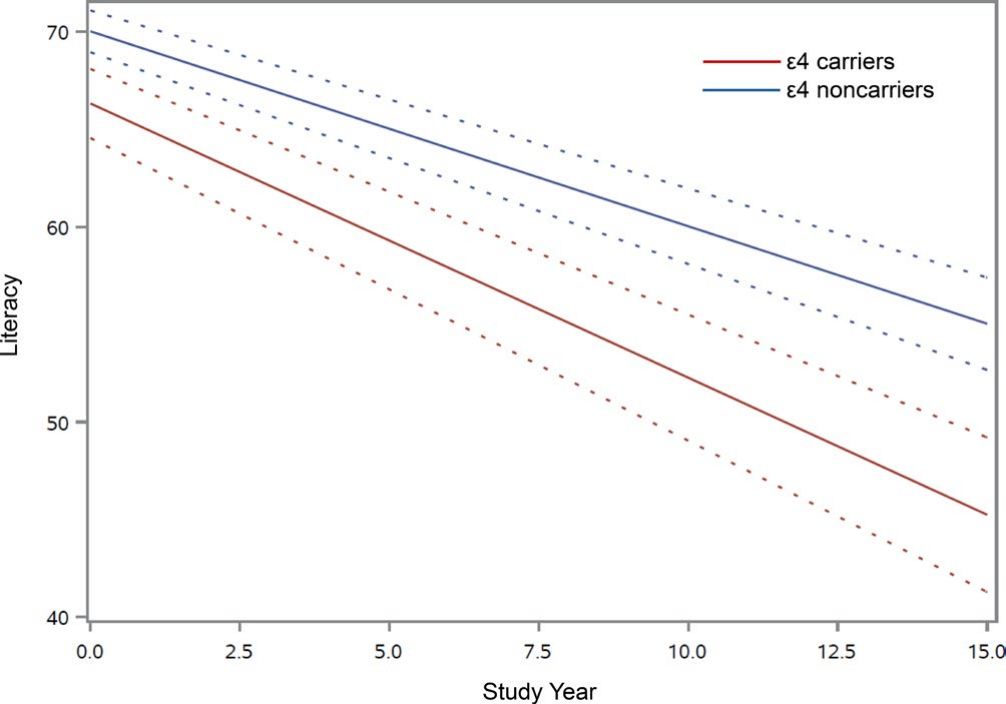
Model‐predicted starting level of literacy and 15‐year paths of change in literacy for participants who were ε4 carriers (red line) and noncarriers (blue line), with 95% confidence bands, adjusted for age, gender, education, and their interactions with time. The possible range of literacy scores is 0%–100%.

Please refer to Table 1 for descriptive characteristics of the participants. During up to 14 years of annual follow‐up (*M* = 7.7, SD = 3.8), 271 of 851 participants (31.8%) developed Alzheimer's dementia, and literacy declined an average of 1.1 percentage points per year (*b* = −1.10, SE *b* = 0.07, *p* < 0.001). In the core model, ε4 was associated with a lower starting level of literacy (*b* = −3.60, SE *b* = 1.00, *p* < 0.001) and, critically, a steeper decline in literacy (*b* = −0.41, SE *b* = 0.14, *p* = 0.004; Table 2, Model A). The effect of ε4 on literacy decline was substantial. In absolute terms, ε4 corresponded to an additional loss of 0.4 percentage points per year. In relative terms, ε4 corresponded to a roughly 40% steeper literacy decline compared to noncarriers (Figure 1) or to the literacy decline associated with being about 6 years older (for full model results, please see Table S1 in the Supporting Information). In supporting analyses, the association of ε4 with steeper literacy decline persisted after additionally adjusting for global cognition at analytic baseline (*b* = −0.35, SE *b* = 0.14, *p* = 0.012; Table 2, Model B) and when analyses were restricted to participants with no cognitive impairment at baseline (*b* = −0.34, SE *b* = 0.14, *p* = 0.016; Table 2, Model C).

**TABLE 1:** Descriptive characteristics of participants at analytic baseline.

Characteristic	*M* (SD) or percent
Literacy (possible range: 0%–100%)	68.2% (14.4)
*APOE* ε4 carrier	22.1%
Age	81.2 (7.5)
Male	23.0%
Education	15.3 (2.9)
Global cognition	0.19 (0.5)
MMSE (possible range: 0–30)	28.3 (1.7)
No cognitive impairment	79.4%

Abbreviations: *M*, mean; MMSE, Mini‐Mental State Examination; SD, standard deviation.

**TABLE 2:** Association of *APOE* ε4 with starting level of literacy and change in literacy.

Model	Model term	*b* (SE *b*)	*p*
A (core)	*APOE* ε4	−3.60 (1.00)	< 0.001
	Time	−1.00 (0.08)	< 0.001
	*APOE* ε4 × time	−0.41 (0.14)	0.004
B (adjusted for cognition)	Global cognition	14.73 (0.74)	< 0.001
	*APOE* ε4	−2.01 (0.83)	0.016
	Time	−1.18 (0.09)	< 0.001
	Global cognition × time	0.65 (0.13)	< 0.001
	*APOE* ε4 × time	−0.35 (0.14)	0.012
C (no cognitive impairment)	*APOE* ε4	−2.51 (1.08)	0.021
	Time	−0.82 (0.08)	< 0.001
	*APOE* ε4 × time	−0.34 (0.14)	0.016

*Note:* All models adjusted for age, gender, and education and their interactions with time.

Abbreviations: *b*, unstandardized beta; *SE b*, standard error for unstandardized beta.

## 4 Discussion

This study examined the association between *APOE* ε4 and change in literacy over a mean of 7.7 years among 851 older adults without dementia. In a linear mixed‐effects model adjusted for age, gender, and education, ε4 was associated with a lower starting level of literacy and, more importantly, an approximately 40% steeper decline in literacy. The association between ε4 and literacy decline persisted after further adjusting for a robust measure of global cognition and among a subgroup of participants with no cognitive impairment at baseline. These findings provide compelling evidence that ε4 exerts a substantial negative impact on literacy, a resource that is not typically directly assessed yet is central to older adults' everyday functioning.

This study raises several clinically‐relevant issues. First, a formidable literature has associated low literacy with adverse outcomes in aging, including poorer health [3, 4], worse financial standing [5, 6, 7], and death [8], and the current findings raise the possibility that *APOE* ε4 helps explain these associations. Second, our findings provide compelling evidence that literacy is susceptible to pathologic brain aging and that literacy decline may forecast impending adverse cognitive outcomes [22]. This runs contrary to the notion that literacy usually remains stable in aging and suggests specific points of intervention (e.g., targeting psychosocial factors that bolster brain reserve and buffer against common aging‐related neuropathologies [23]). It also suggests that literacy might be an important behavioral outcome in clinical trials, as literacy appears to be sensitive to pathologic brain aging and carries real‐world meaning to patients, families, and policymakers. Third, in prior work, we demonstrated that literacy decline is more potently related to deleterious psychological and financial outcomes than the starting level of literacy [11]. Given this, and with older adults increasingly seeking or obtaining *APOE* genotyping on their own, this study provides new motivation for clinicians to discuss long‐term planning (e.g., advanced directives, estate planning) with patients who are ε4 positive.

This study does not address underlying mechanisms, but we posit that two factors help explain the association between ε4 and literacy decline. The first is that ε4 is associated with the accumulation of Alzheimer's disease pathology (i.e., beta amyloid and tau tangles) [24] and DNA‐binding protein of 43 kDa (TDP‐43) [25], neuropathologies that we previously associated with lower literacy [22, 26]. The second factor involves the complexity of literacy. Behaviorally, literacy requires one to seek out, learn, and integrate new health and financial knowledge with old knowledge and apply this updated knowledge in accordance with one's unique life circumstances. Neurologically, literacy likely arises from the coordinated activity of several large‐scale neural networks, including frontosubcortical networks involved in motivation and planning (which support information seeking) [27], temporoparietal networks involved in semantic processing (which support updating of health and financial knowledge) [28], and frontoparietal networks involved in problem solving (which support the application of health and financial knowledge to specific situations) [29]. This behavioral and neurological complexity might render literacy vulnerable to ε4‐related neuropathological processes and might explain why ε4 was associated with lower literacy even at baseline. In addition, about one‐third of participants developed Alzheimer's dementia during follow‐up, and incident dementia may play a role in explaining the association between ε4 and literacy decline as well. In future work, we plan to delve deeper into the precise time course of ε4‐related literacy decline and underlying neuropathologic pathways.

This study has strengths and limitations. Its main strength is the annual assessment of literacy for up to 14 years in a group of well‐characterized older adults. This allowed us to precisely quantify person‐specific slopes of change in literacy, ensure that participants did not have dementia at baseline, and account for potential confounding variables. One weakness is that, while the current literacy measure has been associated with a range of relevant behaviors and outcomes, it is still a proxy for how older adults engage with, learn about, and apply health and financial knowledge in the real world. A second limitation is that participants were predominantly White. The frequency of *APOE* alleles varies by ancestry, and the relation of ε4 with neuropathology and adverse cognitive outcomes may vary by ancestry [30]. Our group assesses literacy on an annual basis among older adults who are African American through the Minority Aging Research Study (MARS), and we can examine the relation of ε4 with literacy in these participants once we have sufficient data. Another important area of future research is to more fully characterize the clinical significance of change in literacy in aging (vs. level of literacy).

## Author Contributions

Study conceptualization, data analysis, interpretation, and drafting of the manuscript: Stewart, Yu, and Boyle. Study conceptualization, study design, data acquisition, and interpretation of findings, and critical review of the manuscript: Kapasi and Bennett.

## Funding

This work was supported by the National Institute on Aging (R01 AG017917 to D.A.B. and R01 AG033678, R01 AG034374, and R01 AG060376 to P.A.B.).

## Conflicts of Interest

The authors declare no conflicts of interest.

## Supporting information


**Table S1:** Full results of linear mixed‐effects models.
