# Supplementary material for: APOE ε4 and Decline in Health and Financial Literacy in Advanced Age
Source: J Am Geriatr Soc. 2025 Dec 30;74(4):1121–5. doi: 10.1111/jgs.70291 (PMC13080071; doi:10.1111/jgs.70291)
Supplement: Supplementary file 1 — Table S1: Full results of linear mixed‐effects models. [file JGS-74-1121-s001.pdf]

Table S1

Full results of linear mixed-effects models

Model	Model term	*b* (*SE b*)	*p* value
A (core)	Intercept	70.0 (0.55)	<.001
	Time	-1.00 (0.08)	<.001
	Age	-0.52 (0.06)	<.001
	Male	4.83 (1.00)	<.001
	Education	1.62 (0.14)	<.001
	APOE ε4	-3.60 (1.00)	<.001
	Age x time	-0.07 (0.01)	<.001
	Male x time	0.20 (0.14)	.15
	Education x time	-0.02 (0.02)	.37
	APOE ε4 x time	-0.41 (0.14)	.004
B (adjusted for cognition)	Intercept	65.9 (0.50)	<.001
	Time	-1.18 (0.09)	<.001
	Age	-0.19 (0.05)	<.001
	Male	6.11 (0.82)	<.001
	Education	0.80 (0.13)	<.001
	Global cognition	14.73 (0.74)	<.001
	APOE ε4	-2.01 (0.83)	.016
	Age x time	-0.06 (0.01)	<.001
	Male x time	0.21 (0.14)	.12
	Education x time	-0.05 (0.02)	.021
	Global cognition x time	0.65 (0.13)	<.001
	APOE ε4 x time	-0.35 (0.14)	.012

Note. b, unstandardized beta. *SE b*, standard error for unstandardized beta.

Table S1 (cont.)

Full results of linear mixed-effects models

Model	Model term	*b* (*SE b*)	*p* value
C (no cognitive impairment)	Intercept	71.5 (0.58)	<.001
	Time	-0.82 (0.08)	<.001
	Age	-0.41 (0.06)	<.001
	Male	4.61 (1.07)	<.001
	Education	1.51 (0.15)	<.001
	APOE ε4	-2.51 (1.08)	.021
	Age x time	-0.06 (0.01)	<.001
	Male x time	0.07 (0.14)	.60
	Education x time	-0.01 (0.02)	.78
	APOE ε4 x time	-0.34 (0.14)	.016

Note. b, unstandardized beta. *SE b*, standard error for unstandardized beta.
